# An Adaptive Infrared Small-Target-Detection Fusion Algorithm Based on Multiscale Local Gradient Contrast for Remote Sensing

**DOI:** 10.3390/mi14081552

**Published:** 2023-08-02

**Authors:** Juan Chen, Lin Qiu, Zhencai Zhu, Ning Sun, Hao Huang, Wai-Hung Ip, Kai-Leung Yung

**Affiliations:** 1 Innovation Academy for Microsatellites of Chinese Academy of Sciences, Shanghai 200120, China; qiul@microsate.com (L.Q.); zczhu@hotmail.com (Z.Z.); sunn@microsate.com (N.S.); 2 University of Chinese Academy of Sciences, Beijing 100000, China; 3 Hubei Key Lab of Ferro & Piezoelectric Materials and Devices, Faculty of Physics and Electronic Science, Hubei University, Wuhan 430062, China; 4 Department of Industrial and Systems Engineering, The Hong Kong Polytechnic University, Hong Kong 100872, China; wh.ip@polyu.edu.hk (W.-H.I.); kl.yung@polyu.edu.hk (K.-L.Y.); 5 School of Engineering, University of Saskatechewan, Saskatoon, SK S7K 0C8, Canada

**Keywords:** IR small target detection, local gradient contrast, target detection

## Abstract

Space vehicles such as missiles and aircraft have relatively long tracking distances. Infrared (IR) detectors are used for small target detection. The target presents point target characteristics, which lack contour, shape, and texture information. The high-brightness cloud edge and high noise have an impact on the detection of small targets because of the complex background of the sky and ground environment. Traditional template-based filtering and local contrast-based methods do not distinguish between different complex background environments, and their strategy is to unify small-target template detection or to use absolute contrast differences; so, it is easy to have a high false alarm rate. It is necessary to study the detection and tracking methods in complex backgrounds and low signal-to-clutter ratios (SCRs). We use the complexity difference as a prior condition for detection in the background of thick clouds and ground highlight buildings. Then, we use the spatial domain filtering and improved local contrast joint algorithm to obtain a significant area. We also provide a new definition of gradient uniformity through the improvement of the local gradient method, which could further enhance the target contrast. It is important to distinguish between small targets, highlighted background edges, and noise. Furthermore, the method can be used for parallel computing. Compared with the traditional space filtering algorithm or local contrast algorithm, the flexible fusion strategy can achieve the rapid detection of small targets with a higher signal-to-clutter ratio gain (SCRG) and background suppression factor (BSF).

## 1. Introduction

Infrared detectors can be applied to real-time detection systems. They are not affected by weather and light, and they are widely used in infrared guidance for remote sensing [1]. Infrared imaging is currently being used in many scenarios, including military and civil applications. The technology has promoted the development of small- and medium-sized target recognition and detection in infrared images. Tracking and detecting small targets in space has become the focus of infrared image processing. Infrared image noise originates from the detector, optical components, and circuit systems. At the same time, complex and variable sky backgrounds have an impact on infrared imaging. They are susceptible to interference by clutter and sunlight [2]. Infrared images lack clear texture and details. Also, the image contrast is poor. It is not easy to recognize small targets. Traditional spatial domain detection methods are suitable for specific imaging environments. They rely on filter templates, resulting in poor robustness for detecting infrared targets in complex backgrounds [3]. It is difficult to develop a unified framework for small target recognition and detection. Infrared small-target detection utilizes the image’s inherent attributes to enhance the target and suppress the background to achieve the real target [4].

Traditional infrared small-target detection mainly aims at detecting small cooperative targets. When the signal-to-clutter ratio (SCR) is high, detection before tracking is adopted. When the SCR is low, tracking before detection is applied. Traditional small target detection algorithms include spatial filtering, frequency domain filtering, local contrast-based detection, low-rank, and sparse representation. Spatial filtering contains max–median filtering, max–mean filtering, Gaussian filtering, and morphological filtering methods, etc. Frequency domain filtering mainly includes wavelet transform methods and low-pass filtering algorithms, etc. [5]. Spatial filtering has high timeliness and has been widely used in engineering applications. Max–median, max–mean, and morphological filtering can achieve highly reliable target detection with specific targets under specified backgrounds. But they are highly dependent on the template size. They show poor robustness to noncooperative small target detection. And they are sensitive to environmental changes [6].

In local contrast algorithms, researchers have proposed an algorithm based on local contrast measurement (LCM) [7], which can improve the small target detection rate. But it causes the excessive enhancement of high-brightness point noise, resulting in a high false alarm rate. To solve the shortcomings of LCM, researchers have proposed an improved local contrast measurement algorithm (ILCM). It has resolved the problem of the high false alarm rates. However, the robustness is low due to the excessive reliance on the sliding window [8]. The article [9] proposed a new LCM algorithm using a DOG filter (NLCM), which was more robust in complex backgrounds with high noise. However, the method needed to preprocess before the detection and could not adapt to the variable background. The author provided an infrared target detection algorithm based on a high boost-based multiscale local contrast measure (HB-MLCM). It improved the detection effects without complete prior knowledge. Unfortunately, it was time-consuming [10]. Another article proposed a multiscale relative local contrast (RLCM) [11]. It could enhance real IR targets and suppress all types of interferences simultaneously. But the imprecise approximation led to false alarms and missed detection. A further article proposed a small-target detection algorithm based on multiscale patch contrast measurement (MPCM) [12]. Without prior knowledge, the method improved the performance of small target detection. But it could not adaptively select the segmentation thresholds, resulting in a high false alarm rate. A small target detection algorithm using a human visual contrast mechanism and Gaussian difference filter to obtain saliency maps was proposed in [13]. It improved the detection efficiency; yet, it was not suitable for high-noise environments. The author in [14] proposed an infrared weak small target detection algorithm (LHM) that calculated local uniformity and local nonuniformity under different scale templates, which had better robustness for noncooperative small target detection. But the computational complexity was higher. The article [15] proposed an infrared weak and small-target detection algorithm based on multidirectional disparity, which could significantly improve the SCR. But it did not achieve the balance of enhancing the target and suppressing the highlight background edges. Another article proposed an infrared weak and small-target detection method based on the saliency scale space [16]. They combined saliency images with local contrast differences, which was suitable for scenarios with complex backgrounds. A weighted local contrast enhancement algorithm was proposed in [17]. They improved the SCRG and suppressed the background clutter at the same time. However, the computational complexity was high, making it unsuitable for engineering applications. At present, the algorithms of multiscale fusion to detect small targets are widely used in rapid and high-performance small-target detection in infrared images [18,19,20]. These methods have achieved better detection results in specific background environments.

Based on the above research, this paper proposes a fused multiscale local gradient contrast algorithm for detecting real infrared small targets in complex backgrounds. The background includes large areas of high-brightness clouds and ground buildings. The infrared small-target detection under different complex backgrounds uses different fusion strategies. We utilize template filter and gradient contrast to finish the multiplicative fusion with a low SCR. When the SCR was higher, we would first adopt template filtering to attain the saliency map. Then, we utilize the new gradient uniformity to calculate the contrast. The method can suppress the real complex noise and highlight the background edge, which can improve the SCR of the target, the detection accuracy, and the efficiency. Compared with the existing high-performance algorithms, the computational complexity is lower. In addition, it is more adaptable to target detection under different complexity backgrounds, which could be used for engineering applications.

## 2. Materials and Methods

**Figure 1:**
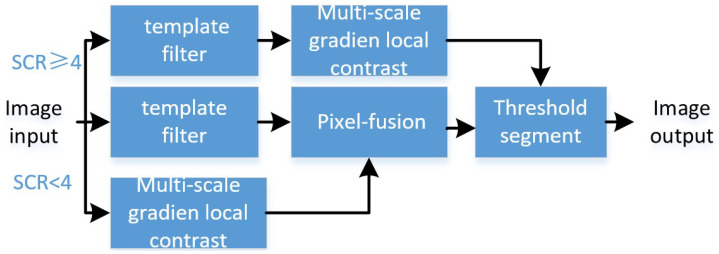
Infrared small-target detection based on adaptive multiscale local gradient contrast.

This article uses a fusion algorithm based on the target’s SCR. SCR stands for the background complexity to some extent [21]. When the SCR was higher than 4, we selected a local gradient contrast enhancement algorithm based on template filtering. First, we used adaptive Gaussian template filtering to obtain the saliency map. The highlight background could be eliminated, but the background edge and the noise remained. Then, we calculated the multiscale gradient contrast based on template filtering. The improved multiscale local gradient contrast defined the gradient uniformity, which is the ratio of the local lowest and the highest contrast. If the contrast ratio of some directions exceeded a certain threshold, the computation value was added to the gradient result. The local contrast uniformity of the background edge was lower than the target, which further strengthened the target and suppressed the background edges and noise. When the SCR was lower than 4, we used the pixel fusion of the multiscale local gradient contrast and template filtering in the original images. It enhanced the target intensity under a relatively complex background. The fusion method could avoid the influence of complex environmental background edges and highlight noise. Finally, after adaptive threshold segmentation, we output the target. The detection strategy is displayed in Figure 1.

### 2.1. A Local Gradient Contrast Enhancement Algorithm Based on Template Filtering

#### 2.1.1. Gaussian Template Filtering

IR sensors can attain images with small targets in the IR target detection system. Every single frame is formed by the background, white Gaussian noise (WGN), and the small target. The mathematical expression is described by (1) [22].

(1) $$f\left(x,y\right)=f_{T}\left(x,y\right)+f_{B}\left(x,y\right)+f_{N}\left(x,y\right)$$

f(x,y) represents the whole IR image. $f_{T}\left(x,y\right)$ is the small target. $f_{B}\left(x,y\right)$ represents the background. The $f_{N}\left(x,y\right)$ is the noise.

Under a typical background with clouds and buildings, the small target displays highlight character. The clouds and buildings are relatively highly correlated with each other. The small target is irrelevant to the background, which occupies the high-frequency band. Because of the distance between the detector and the target, the target could seem like an expansion of the spot target. It can be displayed as (2) [23].

(2) $$f_{T}\left(x,y\right)=\frac{\exp-\frac{\left(\frac{x^{2}}{\delta_{x}^{2}}+\frac{y^{2}}{\delta_{y}^{2}}\right)}{2}}{2\pi \delta_{x}\delta_{y}}$$

Gaussian filtering is applied to preprocess the IR images. It computes a weighted mean in the whole image. Each pixel gray is averaged by the surrounding pixels [24]. The computation utilizes a convolution kernel or template to filter the pixels all over the image. The size of the cooperation targets can be acknowledged beforehand. We can realize effective template filtering with a suitable size for the Gaussian template. However, small targets are mostly unknown in advance. We must create many simulations under the concrete environment with the proper parameters.

Assuming the template is (2k+1)∗(2k+1), the position of the center pixel is (i,j). The Gaussian template can be expressed by (3).

(3) $$G\left(i,j\right)=\frac{\exp-\frac{i^{2}+j^{2}}{2\delta^{2}}}{2\pi \delta^{2}}$$

The target and some highlight edges would remain through choosing the proper template. We can select the most robust template to achieve a better compromise of the target enhancement and the background suppression. If the SCR is relatively high, the Gaussian template filtering could achieve a better saliency map, which contributes to the fusion detection method.

#### 2.1.2. A Local Gradient Contrast Enhancement Algorithm Based on Template Filtering

**Figure 2:**
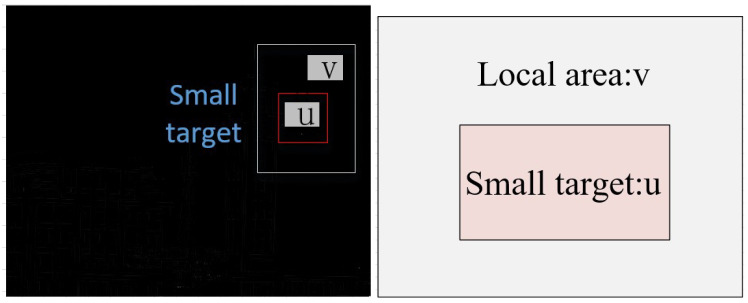
The multiscale local gradient contrast IR small-target detection.

The anisotropic characteristic of the target is different with background edges and Gaussian noise, and the target presents a uniform gradient contrast. The background is continuous with a stationary distribution. The background edge has an uneven gradient contrast. As the size of the local block changes, the local contrast between the target and the background varies. We can define the local surrounding areas *v* and the target *u*. The process is represented in Figure 2.

The target is the base cell named *u*. If the target pixel size is 1∗1, the local patch size is 3∗3. The number of pixels is $N_{u}$. *T* stands for the average pixel gray of the center cell. The mean gray of the target area is defined by (4). $B_{i}$ is the average gray of the *i*th cell surrounding. It is expressed by (5). The local contrast is illustrated by (6).

(4) $$T=mean(I_{j}^{0}).$$

(5) $$B_{i}=\frac{\sum_{j=1}^{N_{u}}I_{j}^{i}}{N_{u}}$$

(6) $$d_{i}=T-B_{i}$$

The local gradient contrast is defined by the gray difference between the target and the surrounding cells in all directions [25]. We define a local gradient contrast based on template filtering as (7).

(7) $$EC\left(x_{i},y_{j}\right)=\min_{i=1,2,3,4}\left(G_{T}-G_{B_{i}}\right)*\left(G_{T}-G_{B_{i+4}}\right)$$

$G_{T}$ and $G_{B_{i}}$ are computed by (3) and (6), respectively. The target, highlight edges, and isolated noise points remain after template filtering. In the same way, the gradient of the targets in each direction is even and consistent in the local area. The gradient in the highlighted background edge moves towards a certain local direction. Its anisotropic gradient is uneven. We should calculate the gradient for all pixels by aggregating all the gradients in all directions. This is displayed in (8).

(8) $$SEC=\sum_{i=1}^{4}EC^{i},\frac{minEC^{i}}{maxEC^{i}}\geq \epsilon .$$

We define the threshold as $\frac{minEC^{i}}{maxEC^{i}}$. The proper parameter ϵ setting is helpful for us to retain the important target details. Then, we compute a local gradient uniformity with (9). *l* is the scale parameter.

(9) $$maxSEC=\max_{l}SEC{\left(x_{i},y_{j}\right)}^{l}.$$

The multiscale operation is good for many noncooperative targets that lack contour information. It is more robust to the complex environment. Finally, we can retain the target without noise through multiscale local gradient contrast.

### 2.2. Adaptive Fusion Algorithm Based on Template Filtering and Multiscale Local Gradient Contrast

Under different complex background conditions, we cannot achieve universal and even detection results. The SCR of the input image represents different background complexities. We selected different fusion strategies depending on the SCR. The local gradient contrast calculation based on a template filter improves the detection effect with a higher detection rate under a lower SCR.

We performed joint detection when the SCR was higher than 4, which is regarded as a relatively clear background. We selected the multiscale local gradient contrast after Gaussian template filtering, which effectively takes advantage of the two methods. The fusion method obtained the balance effect of the background suppression and the target enhancement. When the SCR was lower than 4, we adaptively chose joint detection, which is a pixel fusion of two methods. We adopted pixel fusion, which means the Hadamard product of the template filtering and the multiscale gradient local contrast. It can simultaneously enhance the target and suppress the background under lower SCR conditions, which is helpful for us to detect the target. Selecting the appropriate parameters for adaptive threshold segmentation can achieve better detection results. We attained a set of parameters through simulation and verification in actual IR sequences. Finally, the target was detected with adaptive threshold segmentation under all complex backgrounds.

Compared with the commonly used template detection method and local contrast enhancement method, the algorithm uses the SCR as a priori parameters and utilizes different fusion strategies to realize the rapid detection of small targets under complex environmental backgrounds. A comparison of the advantages and disadvantages with all methods is shown in Table 1.

### 2.3. Evaluation

The most common detection evaluation metrics include *SCRG, BSF*, and the *ROC* curve, which are the quantitative index based on the subject standard. The *SCRG* is shown in (10). It is defined by the SCR of the output image and the SCR of the original input image [2].

(10) $$SCRG=\frac{{\left(S/C\right)}_{o}}{{\left(S/C\right)}_{i}}$$

*S* is the mean difference between the whole image and the small target. *C* is the standard deviation of the whole image. ${\left(\right)}_{i}$ and ${\left(\right)}_{o}$ stand for the parameters of input images and output images.

The BSF is defined by the standard deviation ratio of the input images and output images. It is shown in (11).

(11) $$BSF=\frac{C_{i}}{C_{o}}$$

The ROC curve is composed of the detection rate and the false alarm rate. The detection rate is defined as the ratio of the number of real targets detected to the number of targets in (12).

(12) $$P_{d}=\frac{TP}{T}.$$

TP is the number of real targets detected. *T* is the number of real targets.

The false alarm rate is defined as the ratio of the number of false targets detected to the total number of false targets, as is shown in (13) [21].

(13) $$P_{f}=\frac{FP}{F}$$

FP is the number of false targets detected. *F* is the number of false targets.

The larger *SCRG* and *BSF* indicate better target enhancement and background suppression effect. The *ROC* curve shows the detection rates under all same false alarm rates.

## 3. Results

### 3.1. Real IR Image Attained from the IR Detector System

Our work was based on a real IR detection system that tracked a fast-moving airplane. We used the IR cool mid-wave detector CMS6055 to develop the outdoor experiment. The IR sensor parameters are illustrated in Table 2. It occupied the 3∼5 μm mid-wave infrared band and produced 640∗512 resolution image sequences. The single pixel size was 15 μm. The target was less than 3∗3 pixels according to the detection distance.

**Figure 3:**
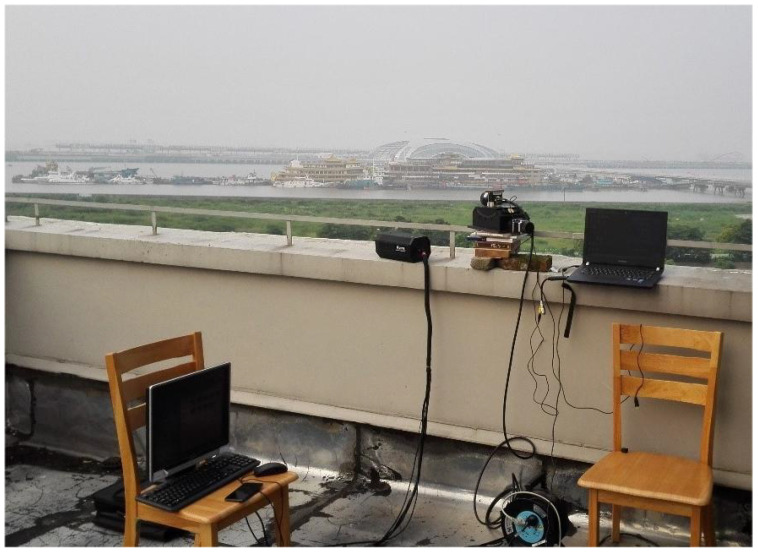
The outdoor IR small-target detection platform.

Under the outdoor platform displayed in Figure 3, we attained three types of IR image sequences. The different algorithms were applied to compare the detection performance of small targets like airplanes under thick cloud and many highlighted buildings’ environments.

### 3.2. Parameter Settings of the Simulation

**Figure 4:**
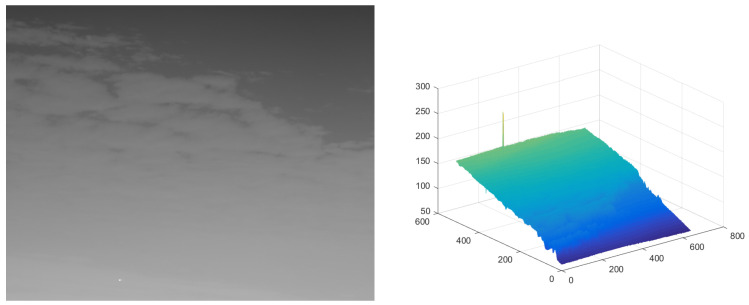
The original image and 3D mesh image of sequence (1).

**Figure 5:**
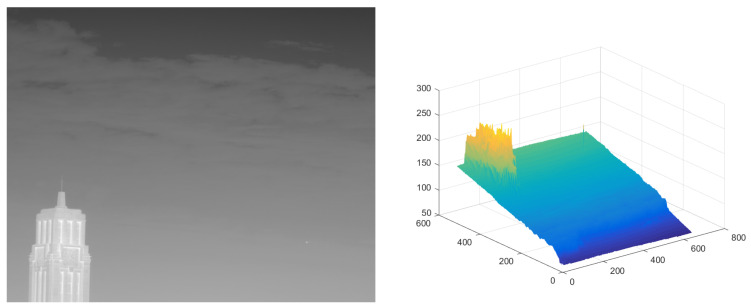
The original image and 3D mesh image of sequence (2).

**Figure 6:**
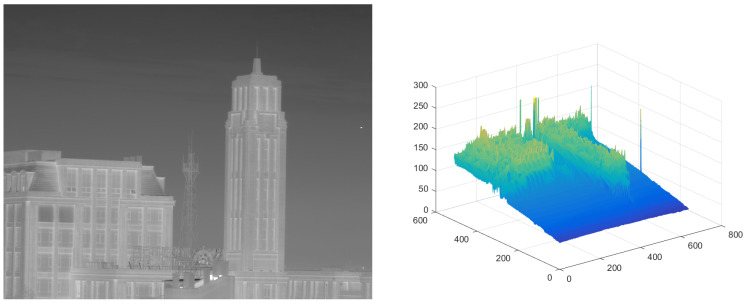
The original image and 3D mesh image of sequence (3).

The test ran on a PC with 16 GB RAM and Intel i7 core CPU. All the algorithms were run in MATLAB 2016b. These image sequences were analyzed with 3D mesh. In Figure 4, the cloud is continuous and has nearly the same gray as the dot target. The second sequence in Figure 5 includes the highlighted buildings, and the target is smaller. It is relatively brighter than the surrounding areas. The third sequence in Figure 6 is complicated, and there are large areas of buildings. The target is barely distinguished from the surrounding areas.

**Figure 7:**
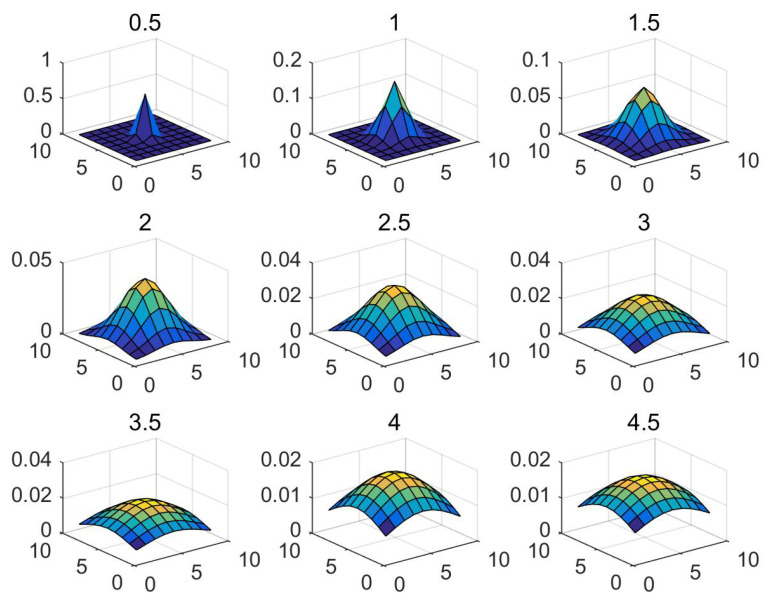
Gaussian Template with different std.

The image sequences achieved by the IR detector were about 60 frames of each kind. With the moving of the target, the character of all elements changed. So, the environment was complicated. We chose the proper parameters such as the standard deviation of the Gaussian template based on many experiments. The template is illustrated in Figure 7. The comparison results are displayed in Table 3.

The effect of Gaussian filtering for different image sequences (a), (b), and (c) is the same after selecting the same standard deviation. As the standard deviation increases, the background suppression effect decreases while the local contrast increases. The detection threshold decreases accordingly. Therefore, the compromise choice of standard deviation is 2 in our data sets.

Then, we selected ϵ as 0.6 in the multiscale local gradient contrast through tests to achieve a better result based on the existing scenarios. With many simulations and analyses, we set the patch size as 3∗3, 5∗5, 7∗7, and 9∗9.

### 3.3. Simulation Results And Comparison

For all sequences with different complexities, we utilized different strategies to strengthen the filtering effects. Then, we used the fusion strategy to finish the process. The SCR of sequence (1) and (2) were less than 4. We selected the local gradient contrast enhancement algorithm based on template filtering. We combined the template filtering with a multiscale local gradient contrast enhancement for sequence (3). The IR images attained a better local contrast and detection rate. The fusion algorithm was much more stable.

**Figure 8:**
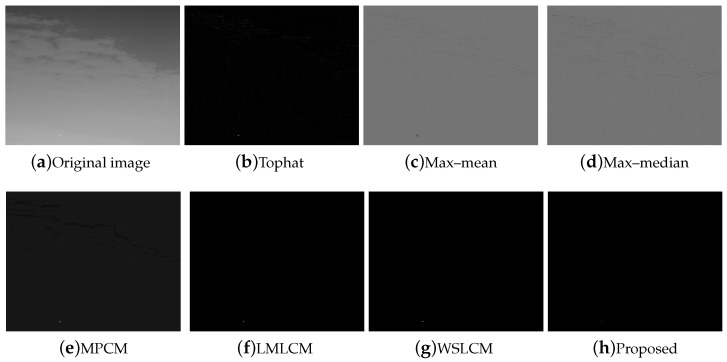
Detection results for different methods in image (1).

**Figure 9:**
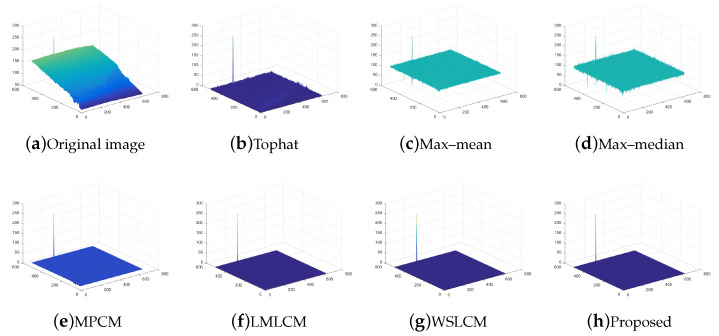
Three-dimensional mesh results for different methods in image (1).

**Figure 10:**
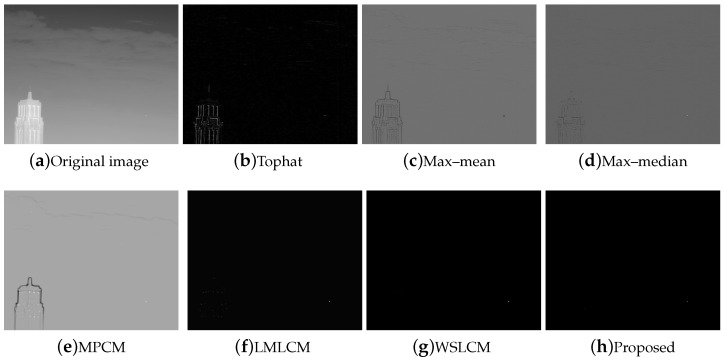
Detection results for different methods in image (2).

**Figure 11:**
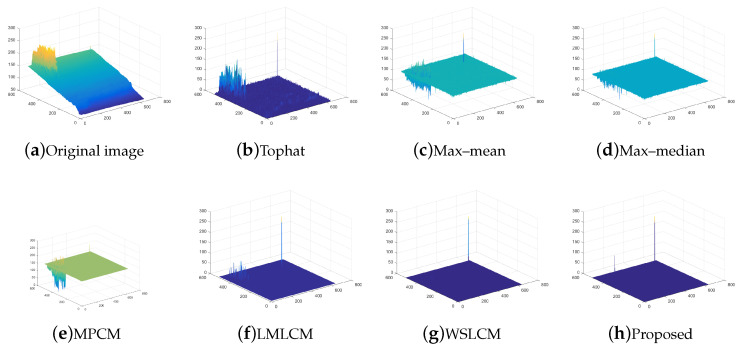
Three-dimensional mesh results for different methods in image (2).

**Figure 12:**
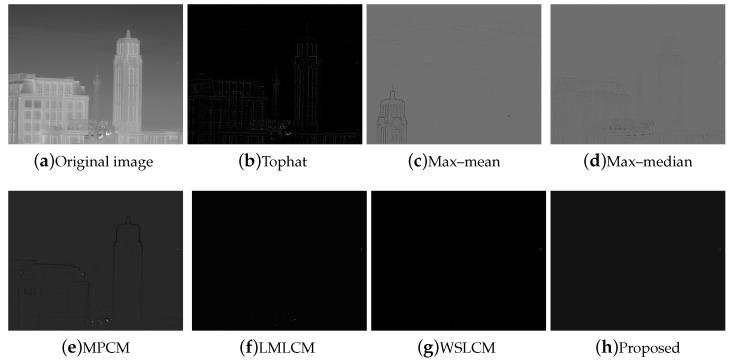
Detection results for different methods in image (3).

**Figure 13:**
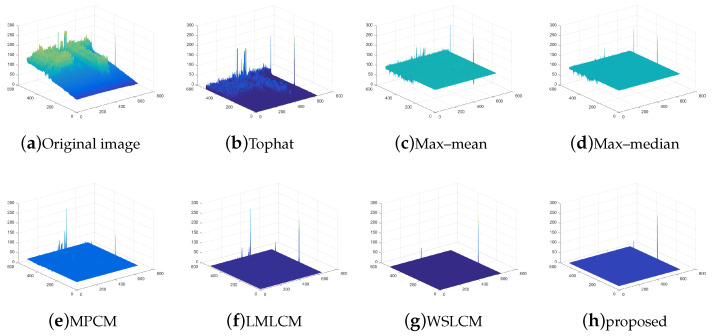
Three-dimensional mesh results for different methods in image (3).

Compared to the other traditional space filtering and contrast measurement methods based on HVS, we achieved a better performance. The figures display the intuitive effect of all the algorithms. The detection comparison of the Tophat, max–mean filter, max–median filter, MPCM [12], LMLCM [20], WSLCM [21], and our method is shown in Figure 8, Figure 9, Figure 10, Figure 11, Figure 12 and Figure 13.

All the methods were compared for the complex background image sequences (1), (2), and (3) captured in the outfield. The SCRG and BSF of image (1) are shown in Table 4. The SCRG and BSF of image (2) are shown in Table 5. The SCRG and BSF of image (3) are displayed in Table 6.

According to the simulations, we set all the parameters in an ideal state. Compared with the traditional algorithms and local contrast algorithms, we used fusion algorithms for the different background sequences (a), (b), and (c). Our proposed algorithm outperformed other commonly used algorithms in terms of the SCRG and BSF except for the WLSCM algorithm. Compared to the WLSCM algorithm, our algorithm had low complexity and greatly reduced the running time for high-resolution real infrared images. The algorithm running time is shown in Table 7.

**Figure 14:**
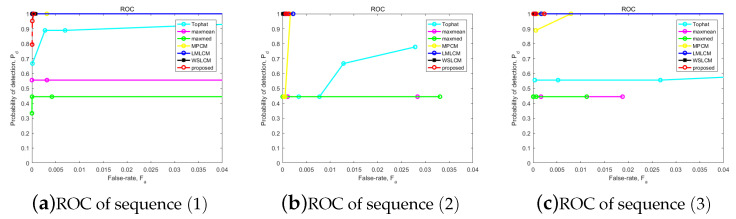
ROC results for the different methods in all sequences.

Finally, we finished the ROC curve. The abscissa was the false positive rate, and the Y-axis was the true positive rate. The ROC curves of the above detection methods are shown in Figure 14. The ROC curve of the proposed strategy showed good performance in the actual infrared image sequences. Our method was better than Tophat, max–mean, max–median, and MPCM. And it was equivalent to the effects of the LMLCM and WLSCM in all sequences. We can conclude that the results indicate that the proposed algorithm has a good robustness and balance effect.

Above all, our method achieved a better SCRG and BSF with less running time. At the same time, we obtained better ROC results. It showed certain equilibrium and stability under complex backgrounds among all metrics.

## 4. Conclusions

The algorithm is an adaptive fusion detection algorithm. The results of the preprocessing have a significant impact on the final detection. While spatial filtering suppresses the background, it also reduces the intensity of the target to a certain extent. It could easily lead to missed detection. At the same time, the local gradient calculation requires selecting an appropriate gradient threshold based on the actual detection image. So, it is necessary to search for more universal templates to achieve the further enhancement of small targets.

The noise of infrared detectors is relatively low due to their cooling effect. The max–median filtering, max–mean filtering, Tophat, MPCM, LMLCM, and WLSCM algorithms and the adaptive fusion algorithm proposed in this article were used for image sequences captured in the real sky environment. The experimental results showed that the Gaussian filtering suppressed the background with a strong correlation. Increasing the gradient contrast at different scales further distinguished small infrared targets, background edges, and white Gaussian noise. The adaptive fusion algorithm was much more suitable to complex environments, effectively improving the background suppression effect with less running time. It can retain a better detection rate under the same false alarm rate.

## Figures and Tables

**Table 1:** Comparison of detection methods.

Method	Advantages	Disadvantages
Tophat	The detection efficiency is high in the environment with low noise and strong target brightness.	It depends on the template size and is insensitive to the environment.
Max–mean	It can suppress pulse noise, pepper and salt noise, and retain the edge details of the image.	It depends on the template size and has a low robustness for environmental complexity.
Max–median	The Gaussian noise is better suppressed when the target is relatively bright and the background is relatively flat.	It has fuzzy edges, a low robustness for environmental complexity, and a low detection efficiency.
MPCM	Bright and dark targets are detected simultaneously, and multiscale computing can be parallel with a high time efficiency.	It has low robustness in a noise environment, and it is easy to cause target separation.
LMLCM	The computational complexity is low, and the low-complexity background detection effect is good.	It is not highly universal and depends on the target template and brightness.
WSLCM	It can achieve a higher reliability ratio gain and a high background suppression effect.	It has high computational complexity and poor timeliness.
Fusion method	In a complex environment, it has a good detection effect and a low calculation complexity, which can achieve good real-time performance.	Only a more balanced detection effect can be obtained; however, the overall optimal effect cannot be obtained.

**Table 2:** IR detector index.

Type	CM6055
detector band	mid-wave 3∼5 μm
pixel size	15 μm
resolution	640 ∗ 512
refrigeration or not	cool
detector size	147 ∗ 94 ∗ 109
weight	1
field of view	$10^{\circ }*8^{\circ }$
aperture and focal length	4 and 55
power supply	less than 25 W 24 V
working temperature	−40 ${}^{\circ }$C + 60 ${}^{\circ }$C

**Table 3:** *BSF* Comparison of IR images sequences.

Std	0.5	1	1.5	2	2.5	3	3.5	4
a	28.87	19.8	17.51	16.23	15.49	15.03	14.75	14.55
b	29.28	20.18	17.86	16.57	15.82	15.36	15.07	14.88
c	29.5	20.3	17.9	16.6	15.8	15.4	15.1	14.9

**Table 4:** Comparison of SCRG and BSF with different detection methods in image sequence (1).

Evaluation	Max-Median	Max-Mean	Tophat	MPCM	LMLCM	WLSCM	Proposed
SCRG	26.704	12.194	8.486	51.595	2392.012	191,796.124	3671.045
BSF	13.870	11.532	7.950	29.154	1237.895	98,974.792	1894.416

**Table 5:** Comparison of SCRG and BSF with different detection methods in image sequence (2).

Evaluation	Max-Mean	Max-Median	Tophat	MPCM	LMLCM	WLSCM	Proposed
SCRG	13.484	11.805	21.664	5.709	118.010	823.409	747.736
BSF	4.657	7.177	12.057	5.571	41.161	281.588	265.267

**Table 6:** Comparison of SCRG and BSF with different detection methods in image sequence (3).

Evaluation	Max-Mean	Max-Median	Tophat	MPCM	LMLCM	WLSCM	Proposed
SCRG	9.689	7.679	11.081	14.513	62.261	294.753	201.51
BSF	5.370	7.505	10.616	17.996	46.431	161.523	119.509

**Table 7:** Comparison results of average running time(s) with different detection methods.

Method	Tophat	Max-Mean	Max-Median	MPCM	LMLCM	WLSCM	Proposed
a	0.180	3.570	3.300	0.277	0.295	23.000	0.300
b	0.050	3.400	3.200	0.227	0.240	25.000	0.280
c	0.040	3.900	3.900	0.225	0.230	24.000	0.250

## Data Availability

The data sets were received from the IR detector. There were about 200 frames of training data. They were representative and covered a complex background.

## Abbreviations

The following abbreviations are used in this manuscript:

IR	Infrared
SCR	Signal-to-clutter rate
WGN	White Gaussian noise
SCRG	Signal-to-clutter rate gain
DBT	Detect before track
TBD	Track before detect
HVS	Human visual system
ROC	Receiver operating characteristic
LCM	Local contrast method
RLCM	Multiscale relative local contrast measure
MLCM	Multiscale local contrast method
ILCM	Improved LCM
MPCM	Multiscale patch-based contrast measure
HBMLCM	High-boost-based multiscale LCM
WSLCM	Weighted strengthened local contrast measure
